# Sample storage-induced changes in the quantity and quality of soil labile organic carbon

**DOI:** 10.1038/srep17496

**Published:** 2015-11-30

**Authors:** Shou-Qin Sun, Hui-Ying Cai, Scott X. Chang, Jagtar S. Bhatti

**Affiliations:** 1 Key Laboratory of Mountain Surface Processes and Ecological Regulation, Institute of Mountain Hazards and Environment, Chinese Academy of Sciences, No. 9, Block 4, South Renmin Rd., Chengdu, 610041 China; 2 Center for Ecological Research, Northeast Forestry University, No.26 Hexing Rd., Xiangfang Dist., Harbin, 150040 China; 3 Department of Renewable Resources, 442 Earth Sciences Building, University of Alberta, Edmonton, AB T6G 2E3, Canada; 4 Canadian Forest Service, Northern Forestry Centre, 5320 122nd St. Edmonton, AB, T6H 3S5 Canada

## Abstract

Effects of sample storage methods on the quantity and quality of labile soil organic carbon are not fully understood even though their effects on basic soil properties have been extensively studied. We studied the effects of air-drying and frozen storage on cold and hot water soluble organic carbon (WSOC). Cold- and hot-WSOC in air-dried and frozen-stored soils were linearly correlated with those in fresh soils, indicating that storage proportionally altered the extractability of soil organic carbon. Air-drying but not frozen storage increased the concentrations of cold-WSOC and carbohydrate in cold-WSOC, while both increased polyphenol concentrations. In contrast, only polyphenol concentration in hot-WSOC was increased by air-drying and frozen storage, suggesting that hot-WSOC was less affected by sample storage. The biodegradability of cold- but not hot-WSOC was increased by air-drying, while both air-drying and frozen storage increased humification index and changed specific UV absorbance of both cold- and hot-WSOC, indicating shifts in the quality of soil WSOC. Our results suggest that storage methods affect the quantity and quality of WSOC but not comparisons between samples, frozen storage is better than air-drying if samples have to be stored, and storage should be avoided whenever possible when studying the quantity and quality of both cold- and hot-WSOC.

The effect of sample storage methods on soil properties being studied is an important issue that needs to be considered before planning an experiment. Although research suggests that soil samples should be analyzed immediately after sampling^1^,^2^, sample storage is not avoidable in many cases for reasons of time limitation or long-distance sample shipping^3^. In such cases, soil samples are commonly air-dried for storage^3^,^4^,^5^ or frozen-stored at −20 °C or lower in a freezer^6^.

Sample storage methods may influence both the physicochemical and biological properties of soils^1^,^7^,^8^,^9^,^10^,^11^. For example, both air-drying and frozen storage may cause a breakdown of soil aggregates^7^. Air-drying has been reported to decrease soil pH and increase extractable Mn, Fe, Cu and Zn contents^8^,^12^, and is considered to be the worst storage method if soil biological properties are to be studied on those stored samples^1^,^10^ as it may cause the death of some bacteria because of hydric stress by the osmotic effects of the drying/rewetting process^9^. Although frozen storage at −20 °C (the most common frozen storage condition) may also change some of the microbial properties, its effects are generally more moderate as compared to that caused by air-drying^10^,^13^,^14^. The Organization for Economic Cooperation and Development^15^ recommended that soil samples should be frozen-stored not exceeding one year if storage in the laboratory is unavoidable. However, some argue that the effect of sample storage methods on soil properties depends on the soil parameter to be studied^10^,^11^.

Soil water soluble organic carbon (WSOC) is the most labile organic C form with fast turnover rates^16^,^17^ and has been extensively studied in recent years due to the important role it plays in C cycling. However, different sample storage methods including air-drying and frozen storage are commonly used prior to sample analysis^3^,^4^,^5^,^6^. These sample storage methods might influence the concentration and chemical property of WSOC. As a result, comparison between studies using different sample storage methods can be difficult.

According to Laura *et al.*^18^, drying would result in the breakdown of organic matter. Air-drying of soil samples has been found to enhance the mineralization of organic matter as air-drying increases the solubilization of organic matter and disrupts soil aggregates^19^. A 3- to 10-fold increase in WSOC was detected in air-dried soil samples in comparison to samples kept in a field moist state^2^,^20^. Zhao *et al.*^21^ found that air-drying increased the WSOC concentration while decreased the difference in WSOC concentrations among soil samples. Even though there are few studies on the effects of air-drying on WSOC and in all these studies the WSOC was extracted by water at room temperature (cold-WSOC), no one has tested the effect of sample storage on WSOC extracted by hot water (70–80 °C, hot-WSOC), and no one has reported the impact of sample storage methods on the changes in the quality of cold- and hot-WSOC. In fact, the quality of WSOC such as the degradability and the aromaticity may be markedly affected by sample storage methods, even if the amount of WSOC is not affected. In addition, few have studied the effect of frozen storage of soil samples on the concentration or quality of both cold- and hot-WSOC, although natural freeze-thaw processes have been found to increase the concentration of simple sugars, carbohydrates and polyphenols in the soil^22^,^23^.

In this study, the concentration and properties of both the cold- and hot-WSOC in various soil types in fresh, air-dried and frozen-stored soil samples were measured. Our objectives were to: 1) test whether air-drying and frozen storage would change the concentration and property of cold- and hot-WSOC in different soil samples; and 2) to explore the relationships between the concentrations of WSOC in fresh soil samples and those in air-dried and frozen-stored samples. We hypothesized that 1) frozen storage would not change while air-drying would increase the concentrations and change the properties of both cold- and hot-WSOC, and 2) there would be a linear relationship in WSOC concentrations between samples stored with different methods as storage methods will cause a systematic shift in the extractability of soil organic C.

## Results

### Concentrations and properties of cold- and hot-WSOC

**Figure 1:**
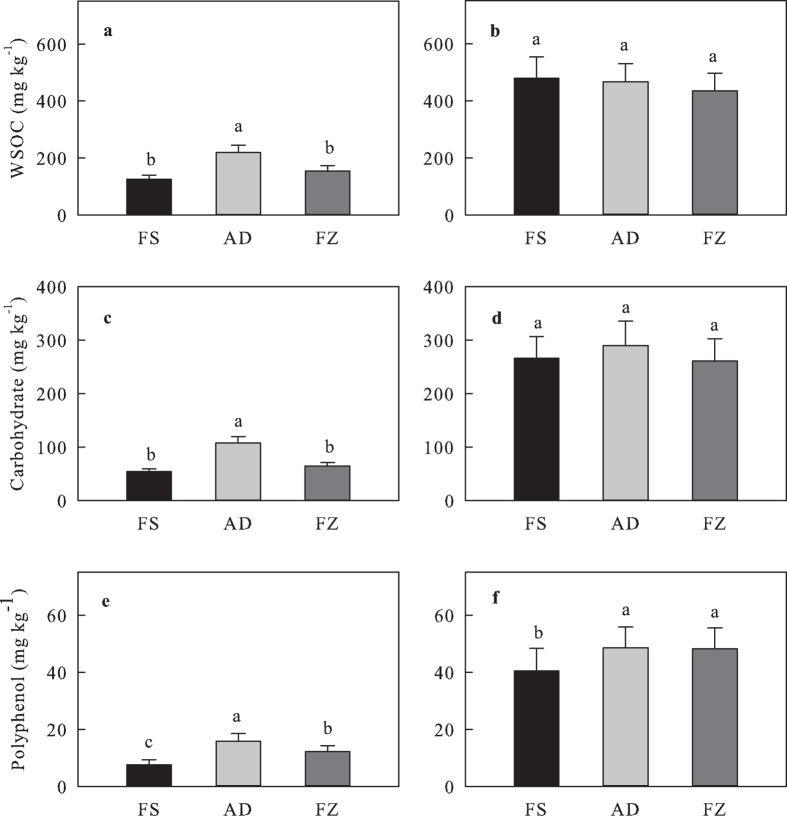
Concentrations of cold- and hot-WSOC and concentrations of carbohydrate and polyphenol in cold- and hot-WSOC (mean ± SE): (a) concentration of cold-WSOC; (b) concentration of hot-WSOC; (c) concentration of carbohydrate in cold-WSOC; (d) concentration of carbohydrate in hot-WSOC; (e) concentration of polyphenol in cold-WSOC; and (f) concentration of polyphenol in hot-WSOC. FS, fresh soil; AD, air-dried soil; FZ, frozen-stored soil; WSOC, water soluble organic carbon.Means with different lowercase letters are significantly different at P < 0.05.

**Figure 2:**
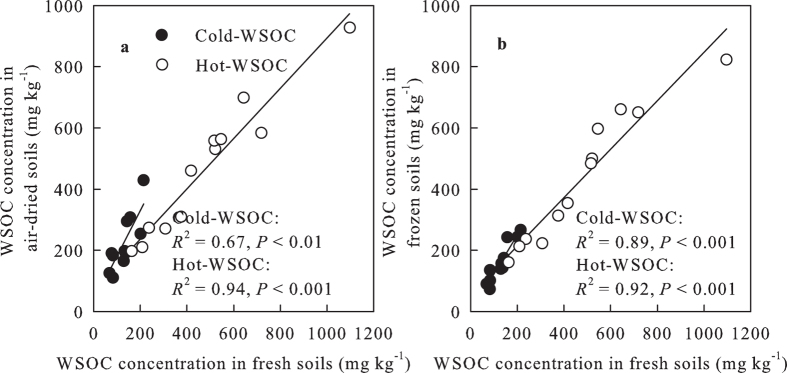
Relationships of cold- and hot-WSOC concentrations between air-dried (AD) and fresh soils (FS) and between frozen-stored (FZ) and fresh soils. WSOC, water soluble organic carbon.

Sample storage methods affected cold-WSOC (*F*_2, 22_ = 28.9, *P* < 0.001) and its carbohydrate concentrations (*F*_2, 22_ = 25.2, *P* <  0.001) (Fig. 1, Table 1). Air-drying resulted in the highest concentrations of WSOC and its carbohydrate concentrations in cold-WSOC. The amount of cold-WSOC in the air-dried soils was linearly correlated with that in the fresh soils (two-tailed test, *R*^2^ = 0.89, *P* < 0.001) (Fig. 2). Frozen storage did not significantly alter the concentration of WSOC and that of carbohydrate in cold-WSOC (Fig. 1a,c). Both air-drying (*P* < 0.001) and frozen storage (*P* = 0.002) increased the concentration of polyphenol in cold-WSOC, with the highest value in the air-dried followed by that in the frozen-stored samples (Fig. 1e). In contrast, neither air-drying nor frozen storage influenced the concentration of WSOC or its carbohydrate concentrations in hot-WSOC (Fig. 1d,e). However, increases in the polyphenol concentration were observed in hot-WSOC in both the air-dried (*P* = 0.001) and the frozen-stored soils (*P* = 0.002) (Fig. 1f).

### Quality of cold- and hot-WSOC

**Figure 3:**
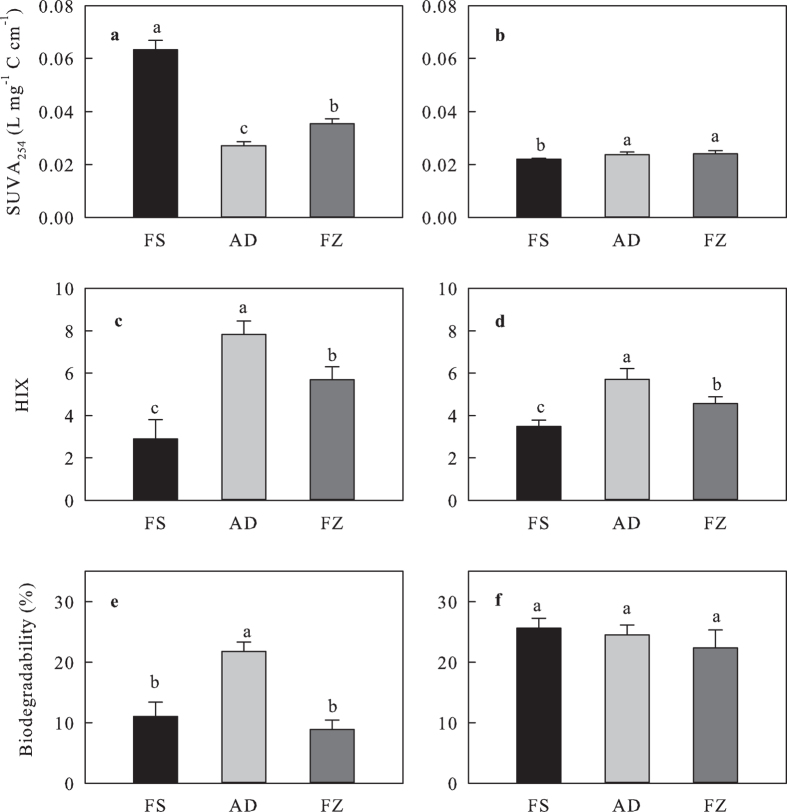
The SUVA_254_ and HIX values and biodegradability of cold- and hot-WSOC (mean ± SE): (a) SUVA_254_ values of cold-WSOC; (b) SUVA_254_ values of hot-WSOC; (c) HIX of cold-WSOC; (d) HIX of hot-WSOC; (e) Biodegradability of cold-WSOC; and (f) Biodegradability of hot-WSOC. FS, fresh soil; AD, air-dried soil; FZ, frozen-stored soil; WSOC, water soluble organic carbon.

Air-drying and frozen storage decreased the specific UV absorbance at 254 nm (SUVA254) of cold-WSOC (*P* < 0.001), with the lowest SUVA_254_ in the air-dried, followed by the frozen-stored and then the fresh samples (Fig. 3a). In contrast, air-drying (*P* = 0.043) and frozen storage (*P* = 0.013) resulted in a significant increase in SUVA_254_ of hot-WSOC (Fig. 3b). The humification index (HIX) in both the cold- and hot-WSOC was significantly increased by air-drying (cold- and hot-WSOC: *P* < 0.001) and frozen storage (cold-WSOC: *P* < 0.001; hot-WSOC: *P* = 0.006), with the highest value in the air-dried soils, followed by the frozen-stored and then by the fresh soil samples (Fig. 3c,d).

The biodegradability of cold-WSOC was significantly influenced by air-drying of samples, where 98.4% increases in biodegradability was observed in the air-dried relative to the fresh samples (*P* = 0.045) (Fig. 3e). However, there was no significant difference among sample storage methods in the biodegradability of hot-WSOC (Fig. 3f).

## Discussion

Air-drying increased while frozen storage at −20 °C in this study did not affect the concentrations of cold-WSOC and carbohydrate in cold-WSOC, supporting part of our first hypothesis. The linear relationships for WSOC concentrations between the air-dried and fresh soils and that between the frozen-stored and fresh soils support our second hypothesis, while it differs with previous results where the difference in cold-WSOC concentration among soil samples was decreased by air-drying^21^ , as well as those reporting that the differences in cold-WSOC concentration between air-dried and fresh soils were proportionately greater for soils with higher total soil organic matter concentrations^20^.

The higher cold-WSOC concentration in air-dried, relative to fresh soils is consistent with results reported by Zsolnay *et al.*^24^, Kaiser *et al.*^25^, Jones & Willett^2^ and Zhao *et al.*^21^. The increased cold-WSOC concentration in air-dried soils may partly come from lysed microbial cells during the drying and rewetting processes that follows (during WSOC extraction)^9^,^21^,^26^. For example, air-drying and the following rewetting process have been found to kill up to ca.70% of the microbial population in soils^27^. The C contained in these dead microbial cells can be rapidly released as soluble organic C when the soil is rewetted^28^. The higher concentration of carbohydrates, one of the main constituents of microbial cells^29^, in the air-dried soils (Fig. 1c) supports the microbial source of the increased cold-WSOC in these soils. In contrast, the unchanged concentrations of cold-WSOC and carbohydrate concentrations in cold-WSOC in frozen-stored soils are likely because microbial cells were preserved when samples were frozen-stored^10^,^13^,^14^,^30^. Although when compared to fresh and frozen samples air-drying may lead to some loss of volatile organic matter from manure where the volatile organic matter concentration is high^31^, we do not expect that to be the case in our soil samples in which the volatile organic matter concentration should be very low.

The WSOC and the carbohydrate concentrations in hot-WSOC were altered neither by air-drying nor by frozen storage, rejecting part of the first hypothesis. This is likely because hot water itself can hydrolyze organic matter, lyse microbial cells, make microbial components extractable^32^,^33^,^34^,^35^,^36^, and dissociate organic materials from inorganic colloids^33^; the sum of those effects together likely eliminates the differences among samples with different storage methods. The result further illustrates that, hot water being a strong extractant that can dissolve a large portion of the soil organic matter, the hot water extraction method is less sensitive for detecting sample storage effects on WSOC. The higher concentrations of polyphenol in both cold- and hot-WSOC in the air-dried and frozen-stored soils relative to the fresh soils were likely caused by the release of humified materials from soil matrixes when samples went through drying/rewetting and freezing/thawing processes, as changes in soil matrixes would increase the release of humified materials^24^.

Although the effect of sample storage such as air-drying on WSOC concentration has been studied, few studied the effect of sample storage on the change in the quality of WSOC as measured by the degree of aromaticity and biodegradability, which is more important in reflecting the stability of WSOC. The aromaticity of WSOC can be measured by the SUVA_254_ index^37^,^38^; the lower SUVA_254_ values in cold-WSOC in the air-dried and frozen-stored soils than in the fresh soils in this study indicate that cold-WSOC was less aromatic and less stable in the air-dried and frozen-stored than in the fresh soils. The HIX provided information about aromatic structures and the complexity of the molecules^39^; the HIX values in cold-WSOC in this study indicate that the air-dried and frozen-stored soils had a higher degree of complexity, conjugation and condensation (i.e., low H/C ratio) of the molecules such as those being variously substituted, condensed aromatic rings, and/or highly unsaturated aliphatic chains^40^,^41^. Contradictory results on the stability of WSOC based on SUVA_254_ vs. HIX were probably because some small-molecular-weight fractions which have low absorbance but high fluorescence were produced during air-drying and frozen storage^42^,^43^. The results also suggest a possibility that part of the aromatic structures in cold-WSOC were broken down and some condensed molecules such as unsaturated aliphatic chains were formed during the air-drying/frozen storage and the wetting/thawing processes that followed. In addition, the evaporation of volatile organic matter during the air-drying processes may change the chemical composition of organic matter in the air-dried soils, resulting in the lowest SUVA_254_ and highest HIX values in those soils relative to the fresh and the frozen-stored soils.

Higher SUVA_254_ values in hot-WSOC in the air-dried and the frozen-stored soils than in the fresh soils were likely because that hot water could extract a wider range of C compounds including phenols, lignin monomers and heterocyclic N-containing compounds^44^, especially when soil aggregates had been physically disrupted by the air-drying/rewetting or the freezing/thawing processes^7^,^24^. Again, the higher HIX indices in hot-WSOC in the air-dried than in the frozen-stored soils show that air-drying and frozen storage promoted the conjugation and condensing of C compounds^24^,^45^.

The higher biodegradability only in cold-WSOC in air-dried soils than in fresh soils in this study is consistent with the elevated carbohydrate concentration in cold-WSOC in the studied soils (Figs 1 and 3). The results are consistent with previous findings that the degradable portion of dissolved organic C is positively correlated with the concentration of carbohydrates^46^,^47^,^48^, and suggest that the effects of air-drying on WSOC properties was greater than frozen storage and cold-WSOC was more sensitive to sample storage methods than hot-WSOC.

## Materials and Methods

### Study site, and soil sampling and processing

To obtain a set of soils representing a range of soil types and properties, 12 soil samples were collected from three sites representing two dominant agroforestry systems (shelterbelt and silvopastural systems) in central Alberta, Canada. In each agroforestry system, there are two land use types, forest (or shrub) and agricultural land uses. The shelterbelt system consists of a variety of trees and shrubs planted in 1–2 rows as shelterbelts^49^ and corresponding agricultural field where the trees and shrubs provide protection against wind and reduce erosion. The silvopasture system consists of grazed aspen forest and the adjacent open pasture where the trees provide shade, shelter and forage for livestock, reducing stress and increasing forage production. Site one (54°35.244′ N and 112°48.204′ W) had a silvopastural system located near Athabasca in north Central Alberta, Canada. The soil type is Dark Gray Chernozem with a loam texture, the mean annual temperature was 2.3 °C and the mean annual precipitation was 469 mm based on data collected between 2009 and 2014 at nearby Atmore AGCM weather station. The trees were dominated by aspen (*Populus tremuloides* Michx.), white birch (*Betula papyrifera* Marsh.), and balsam poplar (*Populus balsamifera* L.), and the dominant herbaceous vegetation was introduced species such as *Bromus inermis* and the system was grazed by cattle in either a rotational or a season-long grazing system. Site two (53°31.793′ N and 113°31.845′ W) was a shelterbelt system located near Camrose in north Central Alberta, Camada. The soil was Black Chernozem with a clay loam texture, the mean annual temperature was 3.7 °C and the mean annual precipitation was 388 mm based on data collected between 2005 and 2014 at nearby University of Alberta Metabolic Centre weather station. The agricultural land was under monocultural annual production system and was converted to agriculture about 100 years ago. Most landowners in the area practice minimum tillage, apply fertilizers, and grow barley (*Hordeum vulgare* L.), wheat (*Triticum aestivum* L.), or canola (*Brassica napus* L.) in rotation. Site three (50°53.996′ N and 111°56.611′ W) was a silvopastural system located at Mattheis Ranch, a microcosm of southern Alberta, Canada, where the land use was grassland and shrubland, the soil type was Brown Chernozem and the soil texture was loamy sand. The mean annual temperature was 4.3 °C and the mean annual precipitation was 319 mm based on data collected between 2005 and 2014 at nearby Rosemary AGDM weather station. The dominant grass species was *Bouteloua gracilis* (H. B. K.) Lag. ex Steud and the dominant shrub was *Shepherdia argentea* (Pursh) Nutt.

Within each site, two paired plots were established in the treed (or shrub) area and its adjacent agricultural land use of the same ecosite, elevation and slope. In each plot, soil samples were collected from the 0–10 and 10–20 cm layers using a soil corer (3.2 cm diameter) in June, 2014, from 20 points along a 100 m transect within each forest- or shrubland-based and adjacent agricultural land use systems. Therefore, four soil samples were collected from each of the three sites, resulting in a total 12 soil samples. The 12 soil samples had different physicochemical properties such as texture, pH, and organic C concentration (Table 2). Soil samples were placed in sealed plastic bags, and kept cool (<4 °C) until they were transported to the laboratory for processing. In the laboratory, samples were sieved (2 mm) to homogenize the sample and to remove visible roots and coarse fragments.

### Experiment design

We used a completely randomized block design to study the effect of three different sample storage methods on the quantity and quality of labile organic C: 1) samples stored at 4 °C and analyzed within 48 hours (fresh, FS); 2) samples were air-dried for two weeks (air-drying, AD) and stored at room temperature until analysis; and 3) samples were frozen-stored in a freezer at −20 °C for one month, and taken out and thawed immediately before measurements (frozen storage, FZ). In the experiment the 12 soil samples served as 12 blocks. Each of the 12 soil samples was divided into three sub-samples and randomly assigned to one of the three treatments. In the laboratory analysis, three previously air-dried soil samples were analyzed together with the FR, AD and FS samples in order to ensure that the data from the two different runs were comparable. The data for those three previously air-dried soil samples were not different between the two runs and thus the data for the experimental samples from the two runs were treated as from the same run.

### Extraction of cold- and hot-WSOC

The cold- and hot-WSOC were extracted and measured according to Li *et al.*^50^. For determining cold-WSOC, a portion of a soil sample equivalent to 15 g oven-dry weight was placed into a 50 mL centrifuge tube, and 30 mL of distilled water was added (soil:water = 1:2, w-v). The centrifuge tube was then shaken at 120 rpm for 30 min at 25 °C, centrifuged for 20 min at 4000 g, and filtered through a 0.2 μm membrane filter (Millipore Corp, USA). For determining hot-WSOC, 15 g of oven-dry equivalent soil samples were placed into 50 mL centrifuge tubes and to each centrifuge tube 30 mL of distilled water was added (soil:water = 1:2, w-v). The centrifuge tubes were then placed in a water bath (80 °C) for 16 h, and then shaken at 120 rpm for 30 min, followed by centrifuging for 20 min at 4000 g. The extract was then also filtered through a 0.2 μm membrane filter (Millipore Corp, USA).

### Analysis of the concentration and quality of cold- and hot-WSOC

The organic C concentrations in cold- and hot-WSOC were analyzed using a Shimadzu TOC-V CSH/CSN analyzer (Shimadzu Corporation, Kyoto, Japan). Subsample of both the cold- and hot-WSOC extracts were analyzed for a) total sugars by the phenol-sulfuric method^51^, using glucose as a standard, after the samples were treated with 0.1 M EDTA (Ethylene diaminetetraacetate), titrated to pH 3.5–4.0 with 5 M KOH and centrifuged at 4000 g for 20 min to prevent co-precipitation of the sugars with cations^52^; and b) total polyphenols by the Folin-Ciocalteu method^53^. A weight/C ratio of 2.5 was used to convert carbohydrates to carbohydrate C, and a ratio of 1.86 was used to convert polyphenols to polyphenolic C^54^.

The cold- and hot-WSOC were then assessed by absorbance and fluorescence analyses on subsamples of cold- and hot-WSOC extracts. The UV–Vis absorbance was measured using a Thermo Spectronic Genesys 10S UV–Visible spectrometer. Samples were placed into a 1 cm quartz cuvette and distilled water was used as the blank. Specific UV absorbance at 254 nm (SUVA_254_), which increases linearly with dissolved organic C aromaticity^37^,^38^, was calculated by dividing the measured absorbance by the concentration of WSOC (L mg C^−1^ cm^−1^).

The fluorescence spectra were obtained with a Photon Technologies International MP-1 spectro fluorimeter (Birmingham, NJ) using quartz cuvettes. Fluorescence emission spectra were collected at an excitation wavelength of 254 nm and an emission wavelength range of 280–500 nm. The humification index (HIX) was calculated as the area under the emission spectra between 435 and 480 nm divided by the sum of the areas between 300 and 345 nm, at 254 nm excitation^55^. The HIX provides information about aromatic structures^39^ and the complexity of the molecules and the degree of humification of soil organic C^56^.

### Biodegradability of cold- and hot-WSOC

To determine the biodegradability of cold- and hot-WSOC, 50 mL of each extract was placed in a clean, acid-washed, 100 mL conical glass flask. All samples were diluted to approximately 10 mg C L^−1^ with distilled water to avoid extensive growth of microorganisms^57^. To each flask, 50 μL of a soil microbial inoculum was added. The soil microbial inoculum was prepared by taking 1 g of the composite soil sample made from all soils and shaking at 20 rpm with 10 mL of distilled water, incubating the suspension for approximately 48 h at 20 °C, and then passing through a 0.45-μm membrane filter. The organic C content of the soil inoculum solution was below detection limit (<0.01 mg L^−1^). No nutrients were added. The flasks were incubated with open tops in the dark at 20 °C for 7 days. The organic C concentration of the samples was measured on days 0 and 7 with the Shimadzu TOC-V CSH/CSN analyzer (Shimadzu Corporation). The day-7 samples were also filtrated through a 0.2 μm membrane filter (Millipore Corp, USA) before analysis in order to remove any potential microbial cells. The incubation was conducted in duplicates. Evaporative losses (<1 g day^−1^) was compensated by adding distilled water every day and before each sampling, to a precision of 0.1 g. Biodegradability was calculated by dividing the difference in organic C concentration between days 0 and 7 by the organic C concentration on day 0.

### Analysis of basic soil properties

Total organic C (TOC) and total nitrogen (TN) concentrations were determined using a Carlo Erba NA 1500 elemental analyzer (Carlo Erba Instruments, Milan, Italy). Soil water content was determined by oven-drying a sub-sample of the soil at 105 °C to constant weight. Soil pH was measured in a suspension of 1:2 soil:water (w:v) using a portable pH meter (PCE Instruments GmbH, Meschede). Soil texture was measured following the hydrometer method described in Kroetsch and Wang^58^.

### Statistical analysis

All data were expressed on an oven-dry soil weight basis. The normality of data was tested with the Shapiro-Wilk test. An analysis of variance (ANOVA) model with the factor block (the 12 soil samples) and treatment (sample storage methods) was used to determine the effect of sample storage methods on the quantity and properties of WSOC. For factors with significant main effects, Tukey’s test was used following the randomized block design ANOVAs to perform multiple comparisons between treatments. Pearson’s correlation analysis with two-tailed test was used to analyze the relationships between the air-dried (AD) and frozen-stored (FZ) soils and the fresh soils for cold- and hot-WSOC concentrations. All analyses were performed with SPSS 19.0 for Windows.

## Conclusions

The concentration of cold-WSOC in soil samples was increased by air-drying but was not affected by frozen storage as compared to the fresh soil, while that of hot-WSOC was not influenced by any of the two storage methods. We conclude that the concentration of cold-WSOC is more sensitive than hot-WSOC to sample storage methods and hot-WSOC is a better estimate of soil labile organic C than cold-WSOC when stored soil samples are to be used for WSOC analysis. Although only air-drying increased the biodegradability of WSOC, both air-drying and frozen storage changed UV specific absorbance and HIX of cold- and hot-WSOC, we therefore conclude that air-drying and frozen storage shifted the molecular structure of WSOC and only evaluating the total WSOC content can be misleading as a lack of sample storage method effect on the concentration does not mean there would be no effect on the properties of WSOC. We suggest that sample storage methods should be selected according to the soil property of concern as the sample storage method effect is soil property-specific. Frozen storage is better than air-drying if samples have to be stored, and storage should be avoided whenever possible when studying the quantity and quality of both cold- and hot-WSOC.

## Additional Information

**How to cite this article**: Sun, S.-Q. *et al.* Sample storage-induced changes in the quantity and quality of soil labile organic carbon. *Sci. Rep.* **5**, 17496; doi: 10.1038/srep17496 (2015).

## Figures and Tables

**Table 1:** ANOVA for the effects of sample storage methods on the concentration and the quality of the cold- and the hot-WSOC.

Dependent variable	Source of variation	Cold-WSOC			Source of variation	Hot-WSOC		
		df	SS	F		df	SS	F
WSOC concentration	Block	11	12595	13.2 ***	Block	11	158557	63.6***
	Treatment	2	27567	28.9***	Treatment	2	6188	2.5
	Residuals	22	955		Residues	22	2492	
Carbohydrate	Block	11	2033	5.3***	Block	11	58272	17.4***
	Treatment	2	9715	25.2***	Treatment	2	2947	0.9
	Residuals	22	386		Residues	22	3351	
Polyphenol	Block	11	154	19.8***	Block	11	2019	87.3***
	Treatment	2	201	25.8***	Treatment	2	251	10.9**
	Residuals	22	7.8		Residues	22	23.1	
SUVA _254_	Block	11	<0.001	3.7**	Block	11	<0.001	7.9***
	Treatment	2	0.004	117.1***	Treatment	2	<0.001	5.6*
	Residuals	22	<0.001		Residues	22	<0.001	
HIX	Block	11	6.9	4.6**	Block	11	4.1	7.2***
	Treatment	2	114	77***	Treatment	2	14.6	25.4***
	Residuals	22	1.5		Residues	22	0.6	
Biodegradability	Block	8	0.01	0.4	Block	11	0.008	1.751
	Treatment	2	0.04	10.8**	Treatment	2	0.003	0.712
	Residuals	16	0.04		Residues	22	0.005	

Significance levels: *P < 0.05; **P < 0.01, ***P < 0.001; WSOC, water soluble organic carbon; SUVA_254_, specific UV absorbance at 254 nm; HIX, humification index.

**Table 2:** Physical and chemical properties (mean ± SD) of the 12 soil samples.

Site location	Soil type	Soil texture	Land use	Sample ID	Soil layer (cm)	TOC (%)	TN (%)	pH
N 54°35.244′ W 12°48.204′	Dark Gray Chernozem	Loam	Grassland	1	0−10	2.59 ± 0.17	0.19 ± 0.04	4.98 ± 0.01
				2	10−20	0.81 ± 0.01	0.04 ± 0.04	5.44 ± 0.04
			Woodland	3	0−10	2.08 ± 0.17	0.15 ± 0.01	4.63 ± 0.01
				4	10−20	0.53 ± 0.12	0.03 ± 0.02	4.63 ± 0.04
N 53°31.793′, W 13°31.845′	Black Chernozem	Clay loam	Cropland	5	0−10	4.05 ± 0.15	0.36 ± 0.01	4.59 ± 0.00
				6	10−20	4.16 ± 0.13	0.33 ± 0.04	5.55 ± 0.01
			Shelterbelt	7	0−10	5.79 ± 0.06	0.47 ± 0.03	5.55 ± 0.01
				8	10−20	4.90 ± 0.16	0.41 ± 0.03	5.23 ± 0.01
N 50°53.996′, W 10°56.611′	Brown Chernozem	Loamy sand	Grassland	9	0−10	1.44 ± 0.01	0.13 ± 0.01	5.76 ± 0.05
				10	10−20	0.74 ± 0.02	0.04 ± 0.00	6.00 ± 0.00
			Shrubland	11	0−10	2.43 ± 0.03	0.20 ± 0.03	5.82 ± 0.01
				12	10−20	1.35 ± 0.06	0.13 ± 0.02	7.36 ± 0.00

TOC and TN represent total organic carbon and total nitrogen, respectively.
